# Exercise Increases Insulin Sensitivity and Skeletal Muscle AMPK Expression in Systemic Lupus Erythematosus: A Randomized Controlled Trial

**DOI:** 10.3389/fimmu.2018.00906

**Published:** 2018-04-27

**Authors:** Fabiana B. Benatti, Cíntia N. H. Miyake, Wagner S. Dantas, Vanessa O. Zambelli, Samuel K. Shinjo, Rosa M. R. Pereira, Maria Elizabeth R. Silva, Ana Lúcia Sá-Pinto, Eduardo Borba, Eloisa Bonfá, Bruno Gualano

**Affiliations:** ^1^Rheumatology Division, Hospital das Clinicas HCFMUSP, Faculdade de Medicina FMUSP, Universidade de Sao Paulo, Sao Paulo, Brazil; ^2^School of Applied Sciences, Universidade Estadual de Campinas (UNICAMP), Limeira, Sao Paulo, Brazil; ^3^Butantan Institute, Sao Paulo, Brazil; ^4^Endocrinology Division, Hospital das Clinicas HCFMUSP, Faculdade de Medicina FMUSP, Universidade de Sao Paulo, Sao Paulo, Brazil

**Keywords:** aerobic exercise, insulin resistance, glucagon, GLUT4, inflammatory rheumatic disease

## Abstract

Systemic lupus erythematosus (SLE) patients may show increased insulin resistance (IR) when compared with their healthy peers. Exercise training has been shown to improve insulin sensitivity in other insulin-resistant populations, but it has never been tested in SLE. Therefore, the aim of the present study was to assess the efficacy of a moderate-intensity exercise training program on insulin sensitivity and potential underlying mechanisms in SLE patients with mild/inactive disease. A 12-week, randomized controlled trial was conducted. Nineteen SLE patients were randomly assigned into two groups: trained (SLE-TR, *n* = 9) and non-trained (SLE-NT, *n* = 10). Before and after 12 weeks of the exercise training program, patients underwent a meal test (MT), from which surrogates of insulin sensitivity and beta-cell function were determined. Muscle biopsies were performed after the MT for the assessment of total and membrane GLUT4 and proteins related to insulin signaling [Akt and AMP-activated protein kinase (AMPK)]. SLE-TR showed, when compared with SLE-NT, significant decreases in fasting insulin [−39 vs. +14%, *p* = 0.009, effect size (ES) = −1.0] and in the insulin response to MT (−23 vs. +21%, *p* = 0.007, ES = −1.1), homeostasis model assessment IR (−30 vs. +15%, *p* = 0.005, ES = −1.1), a tendency toward decreased proinsulin response to MT (−19 vs. +6%, *p* = 0.07, ES = −0.9) and increased glucagon response to MT (+3 vs. −3%, *p* = 0.09, ES = 0.6), and significant increases in the Matsuda index (+66 vs. −31%, *p* = 0.004, ES = 0.9) and fasting glucagon (+4 vs. −8%, *p* = 0.03, ES = 0.7). No significant differences between SLT-TR and SLT-NT were observed in fasting glucose, glucose response to MT, and insulinogenic index (all *p* > 0.05). SLE-TR showed a significant increase in AMPK Thr 172 phosphorylation when compared to SLE-NT (+73 vs. −12%, *p* = 0.014, ES = 1.3), whereas no significant differences between groups were observed in Akt Ser 473 phosphorylation, total and membrane GLUT4 expression, and GLUT4 translocation (all *p* > 0.05). In conclusion, a 12-week moderate-intensity aerobic exercise training program improved insulin sensitivity in SLE patients with mild/inactive disease. This effect appears to be partially mediated by the increased insulin-stimulated skeletal muscle AMPK phosphorylation.

**Clinical Trial Registration:**

www.ClinicalTrials.gov, identifier NCT01515163.

## Introduction

Insulin resistance (IR) is a condition of decreased sensitivity or responsiveness of insulin-sensitive tissues to insulin (1). It is an independent risk factor for type 2 diabetes (T2D) and cardiovascular diseases (CVD), and has been suggested to play a central role in the increased risk of CVD morbidity and mortality in systemic lupus erythematosus (SLE) (2).

We have recently shown that non-T2D SLE patients with mild/inactive disease have increased IR when compared with healthy controls matched by age, gender, body composition, physical activity levels, and food intake (3). Notably, this was despite normal glucose tolerance, as evidenced by normal fasting and postprandial glycemia, as well as preserved skeletal muscle total GLUT4 content and translocation to the membrane in response to a meal. This suggests that SLE patients are capable of overcoming IR in peripheral tissues, preserving glucose uptake at the expense of increased fasting and postprandial insulin secretion, which could lead to β-cell dysfunction and T2D in the long run, increasing CVD risk (4). Patients with SLE also show a state of hyperglucagonemia, both at fasting and postprandially (3). Increased glucagon levels are commonly observed in T2D (5, 6) and have been independently associated with IR in non-T2D individuals (7), suggesting that SLE patients may show at least some level of pancreatic α-cell dysfunction, which could further increase the risk of T2D (8).

Physical exercise is considered a cornerstone for both the prevention and treatment of T2D, as it exerts both acute and chronic beneficial effects on insulin sensitivity (9). A bout of exercise leads to translocation of the glucose transporter GLUT4 from intracellular vesicles to the membrane, facilitating glucose transport into skeletal muscle in an insulin-independent manner (10). Moreover, exercise enhances insulin sensitivity in skeletal muscle, an effect that can last up to 48 h after cessation of exercise (11). If habitually repeated, these bouts of exercise (i.e., exercise training) may further improve insulin sensitivity by enhancing insulin-stimulated glucose transport capacity *via* increases in the expression and/or activity of proteins involved in intracellular insulin signaling pathways, such as protein kinase B (Akt) and AMP-activated protein kinase (AMPK), two important regulators of insulin action in muscle (9, 12).

Exercise has been shown to improve important CVD risk factors in SLE, such as aerobic deconditioning, endothelial function, and autonomic dysfunction (13–17). However, when compared with healthy and T2D individuals, SLE patients are less responsive to the effects of exercise upon lipid profile (18), potentially due to features inherent to the disease, such as persistent inflammation (19). As chronic inflammation can also aggravate IR (20), it is possible that these patients could also be resistant to the beneficial effects of exercise on insulin sensitivity. To our knowledge, there are no studies examining whether, and to what extent, exercise improves IR in SLE and the possible mechanisms involved.

The main aim of the present study was to assess the efficacy of an aerobic training program on insulin sensitivity in SLE patients with mild/inactive disease. Additionally, proteins related to insulin signaling were examined to unravel potential mechanisms underlying exercise-induced improvements in insulin sensitivity in SLE.

## Materials and Methods

### Study Design and Patients

This was a 12-week, randomized controlled trial conducted in the Laboratory of Physical Conditioning for Rheumatologic Patients of the School of Medicine (LACRE), University of São Paulo, Brazil. The manuscript was reported according to the CONSORT statement guidelines. This is the final study that comprised a large clinical trial which aimed to comprehensively investigate the effects of exercise training on the autonomic function, cardiorespiratory parameters, inflammatory markers, and cardiometabolic risk factors in SLE patients (registered at http://clinicaltrials.gov as NCT01515163) (15, 17, 18, 21).

Nineteen adult women with SLE were randomly assigned to participate in a supervised exercise training program (TR; *n* = 9) or to be part of a non-exercised control group (NT; *n* = 10). All SLE patients fulfilled the revised American College of Rheumatology criteria for SLE (22) and were consecutively selected from our outpatient Lupus clinic of the Clinical Hospital at the School of Medicina, University of São Paulo (Hospital das Clinicas HCFMUSP, Faculdade de Medicina, Universidade de Sao Paulo). Disease activity was measured by Systemic Lupus Erythematosus Disease Activity Index (SLEDAI) 2000 scores (23). Exclusion criteria were as follows: age >45 years, body mass index (BMI) ≥35 kg/m^2^, SLEDAI >4, prednisone dose >10 mg/day, menopause, diagnosed T2DM, cardiovascular dysfunction, rhythm and conduction disorders, musculoskeletal disturbances, current kidney and pulmonary involvements, peripheral neuropathy, tobacco use, use of statins, fibrate, insulin or insulin sensitizers, and other systemic autoimmune diseases.

At baseline (Pre), physical activity levels were assessed for characterization purposes using accelerometers, as described elsewhere (24). Patients were strongly instructed to maintain their usual living activities throughout the study. At Pre and after 12 weeks of the intervention (Post), patients underwent a meal test (MT) and were assessed for insulin sensitivity and beta-cell function. Muscle biopsies were performed after the MT for the assessment of total and membrane GLUT4, as well as two candidate proteins related to insulin signaling (Akt and AMPK). Aerobic capacity (using a graded exercise test), body composition [using dual X-ray absorptiometry (DXA)], food intake, and laboratory parameters were also assessed. Post assessments were performed 60–72 h after the last training session in the trained group to avoid any carryover effect associated with the last exercise bout. This study was approved by the Ethics Committee for Analysis of Research Projects of the General Hospital, School of Medicine, University of São Paulo, affiliated to the National Committee for Ethics in Research of Brazil. All subjects signed an informed consent prior to participation.

### Exercise Training Program

Systemic lupus erythematosus patients in the TR group underwent a 12-week, twice-a-week, supervised exercise training program in an intra-hospital gymnasium (Laboratory of Assessment and Conditioning in Rheumatology, School of Medicine, University of São Paulo). The training sessions consisted of a 5-min warm-up followed by 30–50 min of treadmill walking, and a 5-min cooling-down period. The walking duration was gradually increased every 4 weeks, from 30 to 50 min. The intensity of the exercise sessions was set at the heart rate (HR) corresponding to the interval between the ventilatory anaerobic threshold (VAT) and 10% below the respiratory compensation point (RCP), both determined as described in the subsequent subsection.

### Aerobic Capacity

Maximal graded exercise tests were performed on a treadmill (Centurion 200, Micromed, Brazil), with increments in velocity and grade at every minute until volitional exhaustion. Oxygen uptake (VO_2_) and carbon dioxide output (VCO_2_) were obtained through breath-by-breath sampling and expressed as a 30-s average using an indirect calorimetric system (Cortex—model Metalyzer IIIB, Leipzig, Germany). HR was continuously recorded at rest, during exercise and during recovery, using a 12-lead electrocardiogram (Ergo PC Elite, Inc., Micromed, Brazil). Cardiopulmonary exercise test was considered maximal when one of the following criteria were met: VO_2_ plateau (i.e., <150 ml/min increase between two consecutive stages); respiratory exchange ratio value above 1.10; HR no less than 10 beats below age-predicted maximal HR. Peak oxygen consumption (VO_2peak_) was considered as the average of the final 30 s of the test. Ventilatory thresholds were identified as follows: VAT was determined when ventilatory equivalent for VO_2_ (VE/VO_2_) increased without a concomitant increase in ventilatory equivalent for carbon dioxide (VE/VCO_2_), whereas the RCP was determined when VE/VO_2_ and VE/VCO_2_ increased simultaneously.

### Meal Test

After an overnight fast, participants were given a 3-h mixed meal challenge (500 kcal, 60% CHO, 20% fat, and 20% protein). Blood samples were collected at 0, 30, 60, 90, 120, and 180 min for plasma glucose, insulin, proinsulin, triglycerides, and glucagon measurements. Glucose was assessed using a glucose-oxidase method (GOD/PAP, Roche Diagnostics, Germany). Insulin was assessed by a chemiluminescent method (Roche Diagnostics, Germany). Proinsulin and glucagon were assessed by a double-antibody radioimmunoassay (Linco Research, USA). Free fatty acid fasting levels were assessed by a colorimetric assay (Wako, USA). Subjects were instructed to refrain from physical exercise, alcohol, and caffeine intake 24 h prior to the test.

### Surrogates of Insulin Sensitivity

Area under the curve (AUC) of glucose, insulin, glucagon, and proinsulin were calculated using the trapezoidal rule. Whole-body insulin sensitivity surrogate, Matsuda Index, was also calculated from the MT (25); the homeostasis model assessment (HOMA IR) was calculated from fasting glucose and insulin levels (26). Beta-cell function was estimated using the Insulinogenic Index, calculated as the ratio of the incremental insulin and glucose responses at 30 min in response to the MT (27).

### Muscle Biopsies

Muscle samples were obtained from the midportion of the *m. vastus lateralis* of the right limb using the percutaneous needle biopsy technique with suction in a subgroup of patients (SLE-TR, *n* = 4; SLE-NT, *n* = 5) 30 min after the MT. An aliquot of each muscle sample was immediately frozen in liquid nitrogen and stored at −80°C for subsequent analyses.

### Western Blotting

Muscle samples were homogenized and centrifuged in order to isolate the membrane fraction and the cytoplasmatic compartment as previously described (28). Total and membrane fraction protein concentration was determined using the Bradford assay. Samples were subjected to SDS-PAGE in polyacrylamide gel with equal loading (30 µg). Internal loading control was carried out to control for gel-to-gel variability. After electrophoresis, proteins were electrotransferred to a nitrocellulose membrane and monitored with the use of 0.5% Ponceau S staining of the blot membrane. Primary antibodies were incubated overnight at 4°C (GLUT4, 1:1,000, #07-1404, Millipore, Billerica, MA, USA; phospho-Akt Ser 473, 1:1,000, #4058; phospho-AMPK Thr 172, 1:1,000, #2535; GAPDH, 1:1,000, #2118, Cell Signaling Technology, Danvers, MA, USA). Binding of the primary antibody was detected by using peroxidase-conjugated secondary anti-rabbit and mouse antibodies using chemiluminescence (SuperSignal West Femto Chemilumininescent Substrate, Thermo Scientific^®^) detected by ImageQuant LAS 4000 (GE Healthcare^®^) and quantified by densitometry (Scion Image^®^), and normalized to housekeeping (GAPDH). GLUT4 translocation was defined as the ratio of membrane GLUT4 to total GLUT4 (29).

### Body Composition

Body composition (lean, total, and trunk fat mass) was assessed by DXA using Hologic densitometry equipment (Discovery model; Hologic, Inc., USA).

### Food Intake

Food intake was assessed using three 24-h dietary recalls undertaken on separate days (two weekdays and one weekend day) using a visual aid photo album of real foods. Energy and macronutrient intake were analyzed by the Brazilian software Virtual Nutri^®^.

### Statistical Analysis

To minimize the impact of inter-individual variability, all values were converted into delta scores (i.e., Post − Pre values) and thereafter tested by a mixed model, considering Pre values from all dependent variables as covariates. Tukey *post hoc* was used for multiple comparisons. Baseline data were compared using Fisher’s exact tests and unpaired Student’s *t*-tests. Cohen’s *d* was used to determine between-group effect sizes for dependent variables (30). The significance level was previously set at *p* ≤ 0.05, with a trend toward significance being accepted at *p* ≤ 0.1. All analyses were performed using SAS 9.2, SAS Institute Inc., Cary, NC, USA. Data are presented as means ± SDs.

*Post hoc* power analyses were performed with the assistance of the G-Power^®^ software (version 3.1.2) and demonstrated a power of 70 and 60% at an alpha level of 5% to detect significant differences in insulin sensitivity (assessed by the HOMA IR and AUC_insulin_ in response to the MT) between SLE-TR and SLE-NT, with effect sizes (ES) of −1.0 and −0.8.

## Results

### Patients

**Figure 1:**
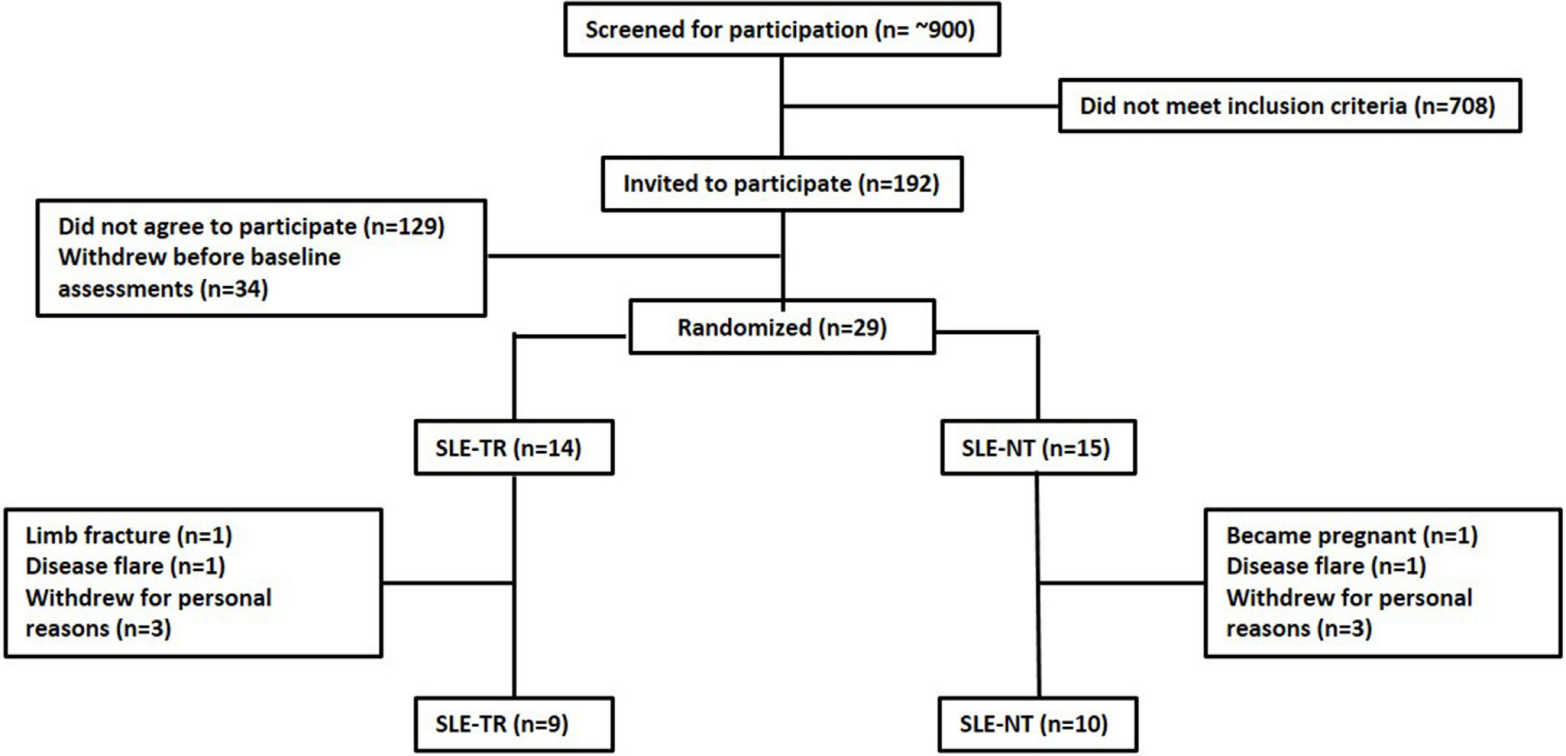
Flow diagram of patients.

Approximately 900 adult SLE patients were initially screened for participation and 192 were eligible. Sixty-three patients initially agreed to take part in the study. Thirty-four patients withdrew from the study before baseline assessments. Thus, 29 patients were randomly assigned to either SLE-TR (*n* = 14) or SLE-NT (*n* = 15). Six patients withdrew from the study for personal reasons (three from SLE-TR and three from SLE-NT), one patient became pregnant (SLE-NT), one patient fractured her limb outside training sessions (SLE-TR), and two patients had disease flare (one from SLE-TR and one from SLE-NT). Thus, the 19 patients who completed the study were analyzed (SLE-TR = 9, SLE-NT = 10) (Figure 1). We opted for a “per protocol” approach instead of an intention-to-treat (ITT) analysis as the primary research goal of our study was to determine the potential efficacy of exercise training on insulin sensitivity and not its effectiveness (31). In this context, ITT analysis has been regarded as more susceptible to type II error (32, 33), as the treatment effect may be diluted due to drop-outs (33). Importantly, baseline comparisons using Fisher’s exact tests and unpaired *T* tests analyses of those who were lost to follow-up and those who retained in each group did not show any drop-out bias (data not shown). Due to technical issues, four patients (one from SLE-TR and three from SLE-NT) were not assessed for glucagon and two patients from SLE-NT were not assessed for proinsulin levels.

Table 1 shows demographic data of the patients. Groups were similar regarding age, BMI, body composition, physical activity levels, current clinical treatment, disease activity status, and disease duration (all *p* > 0.05).

**Table 1:** Demographic, current clinical and treatment data and physical activity levels in SLE patients at baseline.

	SLE-TR (*n* = 9)	SLE-NT (*n* = 10)	CI (95%)	*p*
Age (years)	34.8 ± 4.1	32.4 ± 6.50	−4.8 to 9.5	0.19
BMI (kg/m^2^)	26.3 ± 3.4	26.2 ± 3.8	−4.6 to 4.8	0.94
Total fat (%)	33.5 ± 5.4	34.9 ± 4.5	−8.9 to 6.2	0.60
Trunk fat (%)	30.0 ± 7.3	33.8 ± 7.1	−14.8 to 7.2	0.32
SLEDAI	0.22 ± 0.67	0.40 ± 1.26	−1.3 to 0.9	0.71
Disease duration (years)	9.8 ± 4.1	8.5 ± 5.9	−6.3 to 3.7	0.59

**Drugs**				
Current glucocorticoid use (mg)	1.7 ± 3.5	2.0 ± 4.2	−5.0 to 4.3	0.85
Cumulative glucocorticoid (g/kg)	42.1 ± 31.8	32.4 ± 19.1	−34.8 to 15.4	0.42
Glucocorticoid [no. (%)]	2 (22)	2 (20)	–	1.0
Hydroxychloroquine [no. (%)]	5 (56)	7 (70)	–	0.65
Methotrexate [no. (%)]	2 (22)	2 (20)	–	1.0
Azathioprine [no. (%)]	5 (56)	4 (40)	–	0.66
Mycophenolate [no. (%)]	1 (11)	2 (20)	–	1.0
Cyclophosphamide [no. (%)]	0 (0)	0 (0)	–	1.0
Oral contraceptive [no. (%)]	6 (67)	6 (60)	–	1.0

**Physical activity level**				
Sedentary time (% of day)	56.2 (9.6)	59.4 (8.4)	−20.3 to 11.7	0.49
Total MVPA (min/day)	29.1 (13.7)	25.4 (17.4)	−18.8 to 29.3	0.65
MVPA (min/day in ≥10-min bouts)	8.6 (7.7)	6.8 (8.5)	−12.3 to 15.5	0.68
Counts/day	607,873 (210,321)	605,455 (185,164)	−207,687 to 376,189	0.45

*Data are expressed as mean ± SD or no. (%) and 95% confidence interval (CI)*.

*SLE, systemic lupus erythematosus; SLE-TR, trained group; SLE-NT, non-trained group; BMI, body mass index; SLEDAI, Systemic Lupus Erythematosus Disease Activity Index; MVPA, moderate to vigorous physical activity*.

*Fisher exact tests and unpaired *T*-tests were used to assess possible differences between SLE groups (SLE-TR vs. SLE-NT)*.

### Surrogates of Insulin Sensitivity

**Figure 2:**
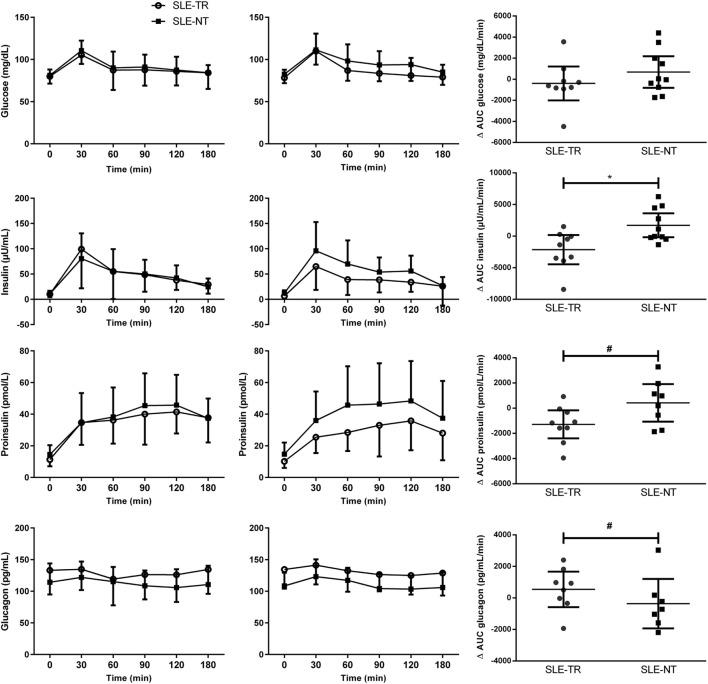
Insulin sensitivity and beta-cell function estimates in SLE-TR and SLE-NT (Pre and Post data and delta changes). Data are expressed as mean ± SD. SLE, systemic lupus erythematosus; SLE-TR, trained group (open circles); SLE-NT, non-trained group (black squares); AUC, area under the curve calculated from the response to the meal test. Mixed model tests adjusted by Pre values were used to assess possible differences in delta changes between groups. *p < 0.05, SLE-TR vs. SLE-NT; ^#^*p* < 0.09, SLE-TR vs. SLE-NT.

Systemic lupus erythematosus-trained group showed, in comparison with SLE-NT, greater decreases in fasting insulin (−39 vs. +14%, *p* = 0.009, ES = −1.0), AUC_insulin_ (−23 vs. +21%, *p* = 0.007, ES = −1.1), HOMA IR (−30 vs. +15%, *p* = 0.005, ES = −1.1), and fasting free fatty acids (−11 vs. +17%, *p* = 0.02, ES = −1.2); a tendency toward decreased AUC_proinsulin_ (−19 vs. +6%, *p* = 0.07, ES = −0.9) and increased AUC_glucagon_ (+3 vs. −3%, *p* = 0.09, ES = 0.6); and greater increases in Matsuda index of whole-body insulin sensitivity (+66 vs. −31%, *p* = 0.004, ES = 0.9) and fasting glucagon (+4 vs. −8%, *p* = 0.03, ES = 0.7). By contrast, no significant differences between SLT-TR and SLT-NT were observed in fasting glucose (−2 vs. +2%, *p* = 0.25, ES = −0.6), AUC_glucose_ (−2 vs. 0%, *p* = 0.28, ES = −0.5), fasting proinsulin (−12 vs. +1%, *p* = 0.39, ES = −0.5), and insulinogenic index (−37 vs. +8%, *p* = 0.23, ES = −0.6) (Table 2; Figure 2).

**Table 2:** Insulin sensitivity and beta-cell function estimates in trained and non-trained SLE patients before and after the exercise intervention.

	SLE-TR (*n* = 9)		SLE-NT (*n* = 10)				
	Pre	Δ (95% CI)	Pre	Δ (95% CI)	Δ diff (95% CI)	*p*	ES
Fasting glucose levels (mg/dL)	79.9 ± 8.20	−1.5 (−5.2 to 2.2)	81.2 ± 9.9	1.4 (−2.1 to 4.8)	−2.8 (−7.9 to 2.2)	0.25	−0.6
AUC_glucose_ (mg/dL)	16,000 ± 2,406	−335 (−184 to 117)	16,425 ± 3,034	73 (−69 to 215)	−1,066 (−3,132 to 1,000)	0.28	−0.5
Fasting insulin levels (μU/mL)	10.0 ± 6.0	−3.9 (−6.7 to −1.1)	10.8 ± 6.0	1.5 (−1.1 to 4.2)	−4.5 (−7.5 to −1.4)	0.009	−1.0
AUC_insulin_ (μU/mL)	8,817 ± 5,638	−2,068 (−3,933 to −204)	8,374 ± 4,589	1,728 (−41 to 3,498)	−3,797 (−6,367 to −1,227)	0.007	−1.1
Fasting glucagon levels (pg/mL)	133.1 ± 38.2	5.9 (−3.3 to 15.2)	114.2 (29.9)	−9.7 (−19.8 to 0.4)	15.6 (1.9 to 29.3)	0.03	0.7
AUC_glucagon_ (pg/mL)	23,108 ± 6,954	681 (−449 to 1,812)	20,166 ± 4,653	−712 (−1,965 to 541)	1,393 (−294 to 3,081)	0.09	0.6
HOMA IR	2.05 ± 1.39	−0.62 (−1.07 to −0.16)	2.21 ± 1.40	0.34 (−0.09 to 0.76)	−0.95 (−1.57 to −0.32)	0.005	−1.1
Matsuda Index	8.1 ± 7.1	5.4 (1.9 to 8.8)	7.3 ± 5.6	−2.3 (−5.6 to 0.9)	7.7 (2.9 to 12.5)	0.004	0.9
Free fatty acids (mEq/L)	0.7 ± 0.3	−0.1 (−0.2 to 0.0)	0.6 ± 0.1	0.1 (−0.0 to 0.2)	−0.2 (−0.3 to 0.0)	0.02	−1.2
Fasting proinsulin	11.3 ± 4.3	−1.4 (−4.0 to 1.2)	14.5 ± 6.0	0.1 (−2.6 to 2.8)	−1.5 (−5.3 to 2.2)	0.39	−0.5
AUC_proinsulin_	6,495 ± 2,220	−1,259 (−2,572 to 53)	6,941 ± 2,831	392 (−919 to 1,704)	−165 (−351 to 20)	0.07	−0.9
Insulinogenic index	3.4 (3.0)	−1.3 (−5.8 to 3.3)	2.9 (2.1)	−0.2 (−1.4 to 0.9)	−1.1 (−2.8 to 0.7)	0.23	−0.6

*Data expressed as mean ± SD. Delta change (Δ) and 95% confidence interval (95% CI), estimated difference between delta changes (Δ difference) and 95% CI, and level of significance (*p*) calculated using a mixed model adjusted by Pre values; effect size (ES)*.

*SLE, systemic lupus erythematosus; SLE-TR, trained group; SLE-NT, non-trained group; AUC, area under the curve calculated from the response to the meal test; HOMA, homeostasis model assessment*.

### Skeletal Muscle Protein Expression and GLUT4 Translocation in Response to the MT

**Figure 3:**
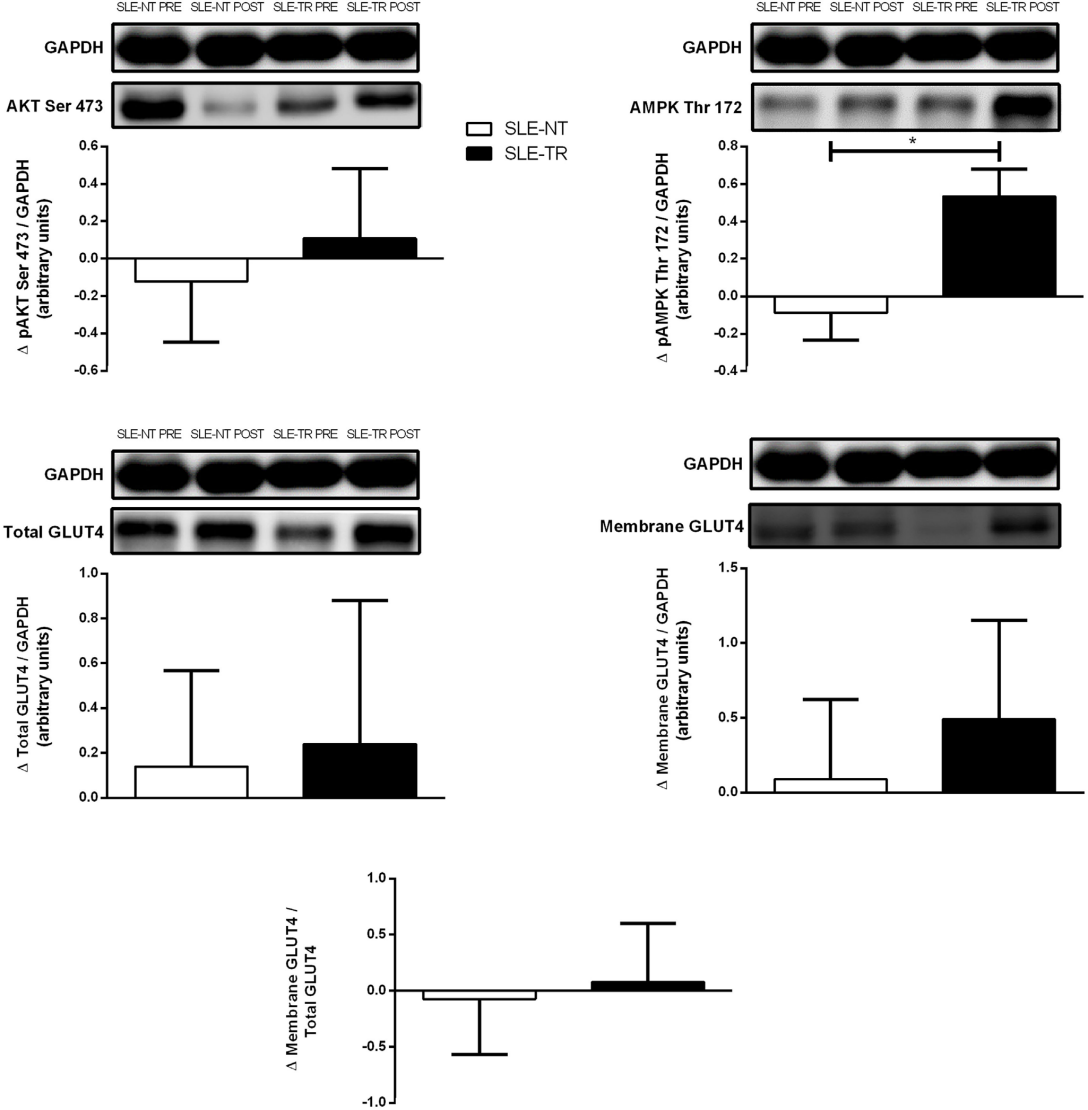
Delta changes for protein expression of skeletal muscle phosphorylated AKT Ser 273, phosphorylated AMP-activated protein kinase (AMPK) Thr 172, total GLUT4, membrane GLUT4, and GLUT4 translocation in response to a meal test in SLE-TR and SLE-NT. SLE, systemic lupus erythematosus; SLE-TR, trained group (*n* = 4); SLE-NT, non-trained group (*n* = 5). Mixed model tests adjusted by Pre values were used to assess possible differences in delta changes between groups. *p < 0.05, SLE-TR vs. SLE-NT.

Systemic lupus erythematosus-trained group showed a significant increase in AMPK Thr 172 phosphorylation when compared with SLE-NT (+73 vs. −12%, *p* = 0.014, ES = 1.3). By contrast, no significant differences between groups were observed in Akt Ser 473 phosphorylation (+16 vs. −12%, *p* = 0.5, ES = 0.7), total GLUT4 expression (+31 vs. +13%, *p* = 0.8, ES = 0.7), membrane GLUT4 expression (+78 vs. +7%, *p* = 0.37, ES = 1.0), and GLUT4 translocation (+9 vs. −5%, *p* = 0.7, ES = 0.6) (Figure 3).

### Aerobic Capacity

Systemic lupus erythematosus-trained group showed significant increases in time at VAT (+35 vs. −6%, *p* = 0.01, ES = 1.2), time at RCP (+21 vs. +10%, *p* = 0.04, ES = 0.9), time to exhaustion (+18 vs. +5%, *p* = 0.008, ES = 1.1), and heart rate peak (+5 vs. +1%, *p* = 0.007, ES = 0.85), whereas no between-group differences were observed in VO_2_peak (+5 vs. +4%, *p* = 0.9, ES = −0.01) (Table 3).

**Table 3:** Body composition, physical capacity, blood parameters, and food intake in trained and non-trained SLE patients before and after the exercise intervention.

	SLE-TR (*n* = 9)		SLE-NT (*n* = 10)				
	Pre	Δ (95% CI)	Pre	Δ (95% CI)	Δ diff (95% CI)	*p*	ES
**Body composition**							
Body weight (kg)	65.0 ± 10.5	−0.3 (−1.7 to 1.1)	67.6 ± 8.8	0.2 (−1.2 to 1.5)	−0.4 (−2.3 to 1.5)	0.6	−0.1
Fat mass (kg)	21.7 ± 6.5	0.1 (−0.9 to 1.1)	22.8 ± 4.8	−0.2 (−1.3 to 0.9)	0.3 (−1.3 to 1.8)	0.7	0.26
Lean mass (kg)	42.0 ± 4.8	0.4 (−1.0 to 1.7)	42.2 ± 5.7	0.3 (−1.1 to 1.8)	0.02 (−1.9 to 2.0)	0.9	0.02
Trunk fat (%)	30.0 ± 7.3	0.4 (−0.7 to 1.4)	33.8 ± 7.1	−0.8 (−1.9 to 0.4)	1.2 (−0.4 to 2.8)	0.13	0.9

**Physical capacity**							
Time at VAT (min)	4.9 ± 1.5	1.7 (0.7 to 2.7)	5.2 ± 0.9	−0.3 (−1.4 to 0.9)	2.0 (0.5 to 3.6)	0.01	1.2
Time at RCP (min)	9.6 ± 1.5	2.0 (1.3 to 2.7)	8.9 ± 1.6	0.9 (0.1 to 1.7)	1.1 (0.0 to 2.1)	0.04	0.92
Time to exhaustion (min)	11.5 ± 1.5	2.1 (1.4 to 2.8)	11.0 ± 1.6	0.6 (−0.2 to 1.4)	1.5 (0.5 to 2.5)	0.008	1.1
VO_2_peak (mL/kg/min)	23.5 ± 4.7	1.1 (−1.2 to 3.4)	22.9 ± 4.6	0.9 (−1.7 to 3.6)	0.2 (−3.3 to 3.7)	0.9	−0.01
HRpeak (bpm)	171 ± 14	9.2 (5.7 to 12.6)	174 ± 11	1.4 (−2.6 to 5.3)	7.8 (2.5 to 13.0)	0.007	0.85

**Blood parameters**							
C3 (90–180 mg/dL)	95 ± 17	−1.6 (−10.9 to 7.7)	101 ± 13	−2.6 (−10.3 to 5.1)	0.9 (−11.2 to 13.1)	0.8	0.26
C4 (10–40 mg/dL)	16.2 ± 4.6	−0.9 (−3.1 to 1.4)	16.8 ± 7.3	−0.3 (−2.1 to 1.5)	1.3 (−3.4 to 2.3)	0.7	0.05
CPK (26–192 U/L)	97 ± 34	−0.5 (−31.3 to 30.2)	114 ± 62	−14.0 (−37.2 to 9.2)	13.5 (−25.0 to 52.0)	0.5	0.44
Creatinine (0.50–0.90 mg/dL)	0.74 ± 0.11	−0.01 (−0.09 to 0.07)	0.67 ± 0.10	−0.01 (−0.07 to 0.06)	−0.00 (−0.11 to 0.10)	0.9	0.24
Urea (10–50 mg/dL)	22.6 ± 4.6	1.0 (−3.4 to 5.5)	27.0 ± 7.2	−1.4 (−4.8 to 2.0)	2.4 (−3.2 to 8.0)	0.4	0.7
Erythrocytes (4.0–5.4 million/mm^3^)	4.0 ± 0.3	−0.1 (−0.3 to 0.1)	4.3 ± 0.2	0.1 (−0.1 to 0.2)	−0.2 (−0.4 to 0.1)	0.14	−0.6
Hematocrit (35–47%)	37.1 ± 1.5	−1.3 (−2.5 to −0.1)	37.6 ± 2.0	0.2 (−0.9 to 1.3)	−1.5 (−3.1 to 0.1)	0.08	−0.7
Leukocytes (4.0–11.0 mil/mm^3^)	4.4 ± 1.6	−0.4 (−1.6 to 0.9)	6.2 ± 2.6	−0.6 (−0.2 to 1.3)	−0.9 (−2.4 to 0.6)	0.2	0.11
Platelets (140–450 mil/mm^3^)	237 ± 49	3.9 (−26.1 to 33.9)	233 ± 49	−0.3 (−27.1 to 26.5)	4.2 (−36.0 to 44.5)	0.8	0.11
CRP (<5 mg/L)	2.3 ± 2.3	−0.3 (−1.9 to 1.3)	3.4 ± 2.8	−0.2 (−1.6 to 1.2)	−0.1 (−2.3 to 1.9)	0.9	0.19
ESR (5.6–11.0 mm)	14.6 ± 12.0	3.1 (−4.3 to 10.6)	15.1 ± 10.4	1.2 (−5.8 to 8.2)	2.0 (−8.2 to 12.2)	0.7	0.22

**Dietary intake**							
Total energy (kcal)	2,022 ± 695	−5 (−696 to 686)	1,885 ± 572	−113 (−769 to 543)	108 (−845 to 1,061)	0.8	0.01
Protein (g)	83.1 ± 35.2	−1.3 (−32.6 to 30.0)	75.3 ± 24.7	1.3 (−28.6 to 31.1)	−2.6 (−45.8 to 40.7)	0.9	−0.15
Protein (%)	20.2 ± 12.6	−4.0 (−6.9 to 1.2)	19.3 ± 9.1	−2.8 (−5.5 to −0.1)	−1.3 (−5.2 to 2.7)	0.5	−0.2
Carbohydrate (g)	258 ± 112	17 (−80 to 113)	239 ± 76	3 (−89 to 96)	13 (−121 to 147)	0.8	0.04
Carbohydrate (%)	47.4 ± 12.6	6.9 (2.7 to 11.1)	47.8 ± 14.8	6.7 (2.8 to 10.7)	0.1 (−5.6 to 5.9)	0.9	0.04
Fat (g)	73.1 ± 28.3	−6.9 (−28.6 to 14.7)	69.8 ± 29.6	−15.6 (−36.2 to 4.9)	8.7 (−21.2 to 38.5)	0.5	0.26
Fat (%)	32.4 ± 7.5	−2.7 (−5.6 to 0.1)	32.9 ± 9.9	−4.2 (−6.9 to −1.5)	1.4 (−2.5 to 5.4)	0.5	0.26

*Data expressed as mean ± SD. Delta change (Δ) and 95% confidence interval (95% CI), estimated difference between delta changes (Δ difference) and 95% CI, and level of significance (*p*) calculated using a mixed model adjusted by Pre values; effect size (ES)*.

*SLE, systemic lupus erythematosus; SLE-TR, trained group; SLE-NT, non-trained group; VAT, ventilatory anaerobic threshold; RCP, respiratory compensation point; VO_2_ peak, oxygen uptake peak; Hrpeak, heart rate peak; CPK, creatine phosphokinase; CRP, C-reactive protein; ESR, erythrocytes sedimentation rate*.

### Body Composition

No significant between-group differences were observed in body weight, fat mass, lean mass, and trunk fat after the intervention (SLE-TR vs. SLE-NT, *p* > 0.05) (Table 3).

### Food Intake

There were no significant differences in total energy or macronutrient intake between groups (*p* > 0.05 for all variables, Table 3).

### Laboratory Parameters

No significant differences in any of the laboratory parameters were observed between SLE-TR and SLE-NT (*p* > 0.05 for all variables; Table 3).

## Discussion

To the best of our knowledge, this was the first study to investigate the efficacy of an exercise training program as well as its potential underlying molecular mechanisms on insulin sensitivity in SLE patients. The main finding of this study was that a 12-week aerobic exercise training program improved insulin sensitivity in patients with mild/inactive SLE. We also identified that this improvement may be associated with increased insulin-stimulated skeletal muscle AMPK phosphorylation, a master regulator of insulin action in muscle.

Systemic lupus erythematosus patients may have increased IR when compared with their healthy peers (3), which is an important risk factor for T2D and CVD (2, 34). In this study, aerobic exercise elicited greater improvements (vs. control) in insulin sensitivity in SLE patients, as reflected by improved HOMA IR (an index based on fasting steady-state parameters which primarily reflects hepatic IR) (26), fasting insulin, insulin response to the MT, and the Matsuda Index (an estimate of whole-body insulin sensitivity derived from the MT) (25). Importantly, these beneficial responses were observed despite no changes in fasting glucose or glucose response to a meal load. These findings were unsurprising because these patients may not have glucose intolerance (3). Thus, the overall improvements in insulin sensitivity should be attributed to the lower secretion of insulin required to maintain glucose levels both at fasting and following nutrient intake. Interestingly, we also observed that fasting free fatty acids levels were diminished after 12 weeks of exercise training, which suggests decreased lipolysis despite lower levels of insulin. Because a major effect of insulin on adipose tissue is to inhibit lipolysis (35), it is likely that insulin sensitivity in adipose tissue was also improved after training. Altogether, these findings suggest that exercise was able to improve hepatic and peripheral insulin sensitivity in SLE patients.

It is noteworthy that improvements in insulin sensitivity in the trained group were shown regardless of any changes in dietary intake or body composition. It is well known that changes in dietary intake, as well as body fat, may impact insulin sensitivity (36–38). Importantly, the current data indicate that exercise *per se*, independent of any concomitant modification in body composition or food intake, can improve insulin action in SLE. It remains to be determined whether interventions resulting in amelioration of eating habits and body fat mass would promote more pronounced enhancements in insulin sensitivity.

The mechanisms by which exercise training enhances insulin sensitivity in skeletal muscle may be associated with increased skeletal muscle GLUT4 content and/or activation of proteins involved in skeletal muscle insulin signal transduction, namely, Akt and AMPK, which can both increase insulin-stimulated GLUT4 translocation (9, 12). To gather some mechanistic insights, we took muscle biopsies and assessed candidate proteins involved in insulin signaling. Although we did not observe any differences in Akt phosphorylation or total skeletal muscle GLUT4 content, we did show a greater increase in AMPK phosphorylation after a meal load in the trained SLE patients. AMPK is a cellular energy sensor and a master regulator of insulin action in muscle. Its activation has been linked to improvements in insulin sensitivity and inflammation (39). However, in the current study, increased AMPK expression did not lead to enhanced GLUT4 translocation, since the higher total GLUT4 expression and membrane GLUT4 expression in trained SLE did not reach statistical significance. The low number of muscle biopsies may have precluded the detection of significant changes. Alternatively, the absence of improvement may be related to the fact that insulin-stimulated GLUT4 translocation is preserved in SLE patients, as previously demonstrated by our group (3). Larger studies are warranted to further clarify the molecular mechanisms underlying the exercise-induced improvement in insulin sensitivity in SLE, as well as the influence of exercise-induced AMPK overexpression upon insulin sensitivity and inflammatory responses.

We have previously shown that SLE patients with mild/inactive disease show preserved beta-cell function as reflected by similar proinsulin-to-insulin ratios and a higher insulinogenic index when compared with their healthy peers (3). The latter surrogate pinpoints the higher insulin secretion needed to overcome IR in SLE patients, which could predispose β-cell dysfunction (4). In the present study, reductions in proinsulin levels paralleled those in insulin levels after the exercise training program, indicating a still preserved beta-cell function. Although the insulinogenic index was reduced in the trained group by 37% (vs. +8% in the non-trained group), this difference did not reach statistical significance. It is possible that the decrease in insulin secretion upon nutrient stimulus after exercise training may not have been enough to incite detectable reductions in the insulinogenic index in the trained group.

We expected any improvements in insulin sensitivity to improve the pancreatic α-cell response to insulin, thus leading to decreased glucagonemia in the trained group. In contrast, exercise training elicited slightly increased glucagon levels at fasting (+4%) and in response to the MT (+3%). This suggests that exercise does not enhance pancreatic α-cell sensitivity to insulin, and that the decreased insulin levels at fasting and in response to a meal load possibly led to a lower suppression of glucagon secretion. We have shown positive correlations between glucagon levels and erythrocytes sedimentation rate (ERS) and interleukin-6 (IL-6) in SLE patients with mild/inactive disease (3), suggesting that these inflammatory markers could partially explain hyperglucagonemia (40). There is evidence that an exercise intervention with similar characteristics to the current one fails to reduce ERS and IL-6 in SLE patients with mild disease (21). Thus, it is possible that greater improvements in systemic inflammation may be necessary to elicit reductions in glucagonemia in these patients, although this emerging hypothesis remains to be examined.

In addition to the positive effects on insulin sensitivity, the exercise training program led to significant improvements in physical capacity, without affecting laboratory and clinical disease-related parameters, in accordance with previous literature (13–16, 21, 41). This further reinforces the notion that exercise is a safe and effective tool in improving CVD risks in mild/inactive SLE. In a clinical setting, therefore, exercise emerges as a valid intervention which should be recommended to treat IR in this disease.

### Limitations

First, we cannot extrapolate our findings to SLE patients with different characteristics, including disease severity, comorbidities, and drug regimens. Regarding the latter, it is well known that some drugs can alter glucose homeostasis (e.g., hydroxychloroquine and glucocorticoid) (42, 43). Since this was a small-scale study, larger trials are necessary to determine whether sub-samples of SLE patients taking specific medications, such as those aforementioned, respond to exercise differently. Second, the methods used to assess insulin sensitivity in this study permitted the calculation of hepatic and whole-body insulin sensitivity estimates, but not the direct assessment of hepatic, peripheral insulin sensitivity, or skeletal muscle glucose uptake. Finally, the small sample size may have limited the power to detect potentially statistically significant changes, particularly for the secondary outcomes.

### Future Directions

This study is the first to demonstrate the value of exercise in improving IR in SLE. *In vitro* and experimental studies are necessary to validate the role of the AMPK pathway and to investigate the role of further canonical pathways not examined in this study (e.g., PPAR-gamma and MAPK) in the improvements of insulin sensitivity in response to exercise in SLE. Moreover, molecular array studies are warranted to explore further potential mechanisms underlying this response, which could lead to potential therapeutic targets. Perhaps more importantly, long-term, well-powered, clinical trials remain necessary to examine the chronic effect of exercise, along with the usual pharmacological treatment, on the prevention of T2DM and CVD in this disease.

In conclusion, a 12-week moderate-intensity aerobic exercise training program can improve insulin sensitivity in patients with mild/inactive SLE. Importantly, this response appears to be associated with increased insulin-stimulated skeletal muscle AMPK phosphorylation.

## Author Contributions

FB, BG, and EB conceived the study. FB analyzed and interpreted the data and drafted the manuscript. CM, WD, VZ, SS, RP, ES AS-P, and EB acquired data. All authors revised and approved the final version of the manuscript and are, thus, accountable for its content.

## Conflict of Interest Statement

The authors declare that the research was conducted in the absence of any commercial or financial relationships that could be construed as a potential conflict of interest.
